# Investigating the impact of 2-OHOA-embedded liposomes on biophysical properties of cancer cell membranes via Laurdan two-photon microscopy imaging

**DOI:** 10.1038/s41598-024-65812-9

**Published:** 2024-07-09

**Authors:** Xuehui Rui, Yukihiro Okamoto, Shuichiro Fukushima, Nozomi Morishita Watanabe, Hiroshi Umakoshi

**Affiliations:** 1 Division of Chemical Engineering, Graduate School of Engineering Science, Osaka University, 1-3 Machikaneyamacho, Toyonaka, Osaka 560-8531 Japan; 2 Department of Mechanical Science and Bioengineering, Graduate School of Engineering Science, Osaka University, 1-3 Machikaneyamacho, Toyonaka, Osaka 560-8531 Japan

**Keywords:** Drug delivery, Lipids, Membrane biophysics

## Abstract

2-Hydroxyoleic acid (2-OHOA) has gained attention as a membrane lipid therapy (MLT) anti-cancer drug. However, in the viewpoint of anti-cancer drug, 2-OHOA shows poor water solubility and its effectiveness still has space for improvement. Thus, this study aimed to overcome the problems by formulating 2-OHOA into liposome dosage form. Furthermore, in the context of MLT reagents, the influence of 2-OHOA on the biophysical properties of the cytoplasmic membrane remains largely unexplored. To bridge this gap, our study specifically focused the alterations in cancer cell membrane fluidity and lipid packing characteristics before and after treatment. By using a two-photon microscope and the Laurdan fluorescence probe, we noted that liposomes incorporating 2-OHOA induced a more significant reduction in cancer cell membrane fluidity, accompanied by a heightened rate of cellular apoptosis when compared to the non-formulated 2-OHOA. Importantly, the enhanced efficacy of 2-OHOA within the liposomal formulation demonstrated a correlation with its endocytic uptake mechanism. In conclusion, our findings underscore the significant influence of 2-OHOA on the biophysical properties of cancer plasma membranes, emphasizing the potential of liposomes as an optimized delivery system for 2-OHOA in anti-cancer therapy.

## Introduction

Plasma membrane fluidity is an important factor that influences cancer cell adhesion and migration. One of the most important properties of cancer cells is altered lipid metabolism, and consequently, abnormal cell membrane composition^1^. The composition and fluidity of the of cancer cell plasma membranes vary across different cell types. For instance, glioma cells exhibit higher fluidity than normal brain cells^2^. Increased cancer cell membrane fluidity correlates with higher metastases rate, while lower fluidity hinders the motility of cancer cells during the epithelial–mesenchymal transition (EMT)^3,4^. Modulation of membrane lipid composition and organization is actively being developed as an effective therapeutic strategy for cancer treatment, and is recognized as membrane lipid therapy (MLT).

2-OHOA (2-hydroxyoleic acid) is the world’s first conditionally approved MLT drug for the treatment of solid tumors^5^. Researchers have focused on 2-OHOA because of its unique ability to activate sphingomyelin synthase (SMS), leading to an abundance of sphingomyelin (SM) content in cell plasma membranes^6,7^. This augmentation of SM levels significantly reduces cancer cell plasma membrane fluidity and prompts the translocation of Ras and the capping of Fas receptor, which subsequently inhibits downstream cell signaling pathways, resulting in the apoptosis and autophagy of cancer cells^8–10^. Exploiting this SMS activation capability, 2-OHOA has been developed for the treatment of solid tumors, with a particular emphasis on its effectiveness in glioma treatment.

Although 2-OHOA is non-toxic anticancer drug, its single-chain lipid structure makes it challenging to dissolve in aqueous solutions. The current method of administering 2-OHOA is through oral delivery, which requires high dosage levels and limits its anti-cancer efficacy. To overcome this obstacle, previous researchers have developed 2-OHOA into various nano drug delivery system (nano-DDS)^11–13^.

To assess the potential of the nano-DDS for delivering 2-OHOA, we embedded 2-OHOA within DOPC-based liposomes. The liposome formulation is a widely utilized nano-drug delivery system, well-researched for its properties and performance. The large inner water pool of liposomes provides high drug loading capacity, potentially facilitating co-delivery of 2-OHOA with other hydrophilic drug payloads^13^. The essential characteristics of the liposomes, including hydrodynamic diameter, ζ-potential and water trapping volume, were investigated. Given that, a significant proportion of nanoparticles tends to accumulate in the liver following intravenous injection due to the clearance of reticuloendothelial system (RES)^14^, and considering the clinical trial focus of 2-OHOA on glioma, this study leveraged both hepatoma (HepG-2) and glioma (NP-8) cells as model cells for 2-OHOA-embedded liposomes treatment. Previous studies have primarily concentrated on elucidating the anti-cancer mechanism of 2-OHOA and exploring the associated cellular signaling pathways^6–8^, without a comprehensive examination of the variations in cell membrane biophysical properties induced by 2-OHOA. This study focused on examining variations in the polarity, fluidity, and lipid packing of cancer cell membranes following treatment with 2-OHOA-embedded liposomes. The overarching objective is to assess the therapeutic efficacy of these liposomes, particularly focusing on the modulation of membrane biophysical properties in the context of 2-OHOA’s anti-cancer potential.

In this study, Laurdan (6-Dodecanoyl-2-Dimethylaminonaphthalene) fluorescence probe was employed to examine variations in cellular membrane polarity/fluidity subsequent to treatment with 2-OHOA-embedded liposomes via two-photon microscopy imaging as well as fluorescence photometer. Laurdan is frequently used as a membrane probe because of its large excited-state dipole moment, which allows it to report the extent of water penetration into the bilayer surface as a result of the dipolar relaxation effect^15^. When solubilized in a lipid bilayer structure, Laurdan senses the environment and its spectrum shifts according to the water content of the bilayer. Water penetration has been correlated with membrane fluidity and lipid bilayer packing^16–18^. Compared to other fluorescent probes used for cell membrane analysis (e.g., DPH, Diphenylhexatriene; Nile Red), Laurdan exhibits dual fluorescence spectrum properties. Its emission spectrum is highly sensitive to changes in membrane phase and polarity. By measuring Laurdan’s fluorescence spectrum, we can distinguish between liquid crystalline and gel phases of membranes. This property is not commonly found in many other fluorescent probes. Additionally, Laurdan’s fluorescence properties facilitate precious quantitative analysis. The generalized polarization (*GP*) value of Laurdan-stained cells was calculated to quantify the cell membrane polarity/fluidity variations. In general, a high *GP* value indicates low membrane polarity or reduced membrane fluidity, whereas a low *GP* value implies the opposite. Microscopy can provide a unique tool for the study of membrane heterogeneity, and two-photon excitation has the additional advantages of inducing less damage to live cells and the significantly decreased photo-bleaching of Laurdan.

Another environment-sensitive fluorescent probes, LipiORDER, was used to corroborate Laurdan imaging results. LipiORDER is a pyren-based solvatochromic fluorescent dye which can be inserted into the lipid bilayer and changes its fluorescent properties in response to the environment^19^. Generally, the liquid ordered (Lo) phase is a highly packed lipid bilayer with low fluidity, whereas the liquid disordered (Ld) phase is a sparsely packed lipid bilayer with high fluidity. Based on the lipid packing of the lipid bilayer membrane, LipiORDER changes its fluorescent color from green on the Lo membrane to red on the Ld membrane. The lipid packing of the cell membrane can be approximated and compared by quantifying the LipiORDER red/green fluorescence intensity ratios (R/G ratio)^20^.

These methodologies enabled the assessment and visualization of alterations in cell membrane polarity, fluidity and lipid packing status, facilitating the evaluation and refinement of various formulations of 2-OHOA-embedded liposomes.

## Result and discussion

### Characterization of 2-OHOA-embedded liposomes

**Figure 1:**
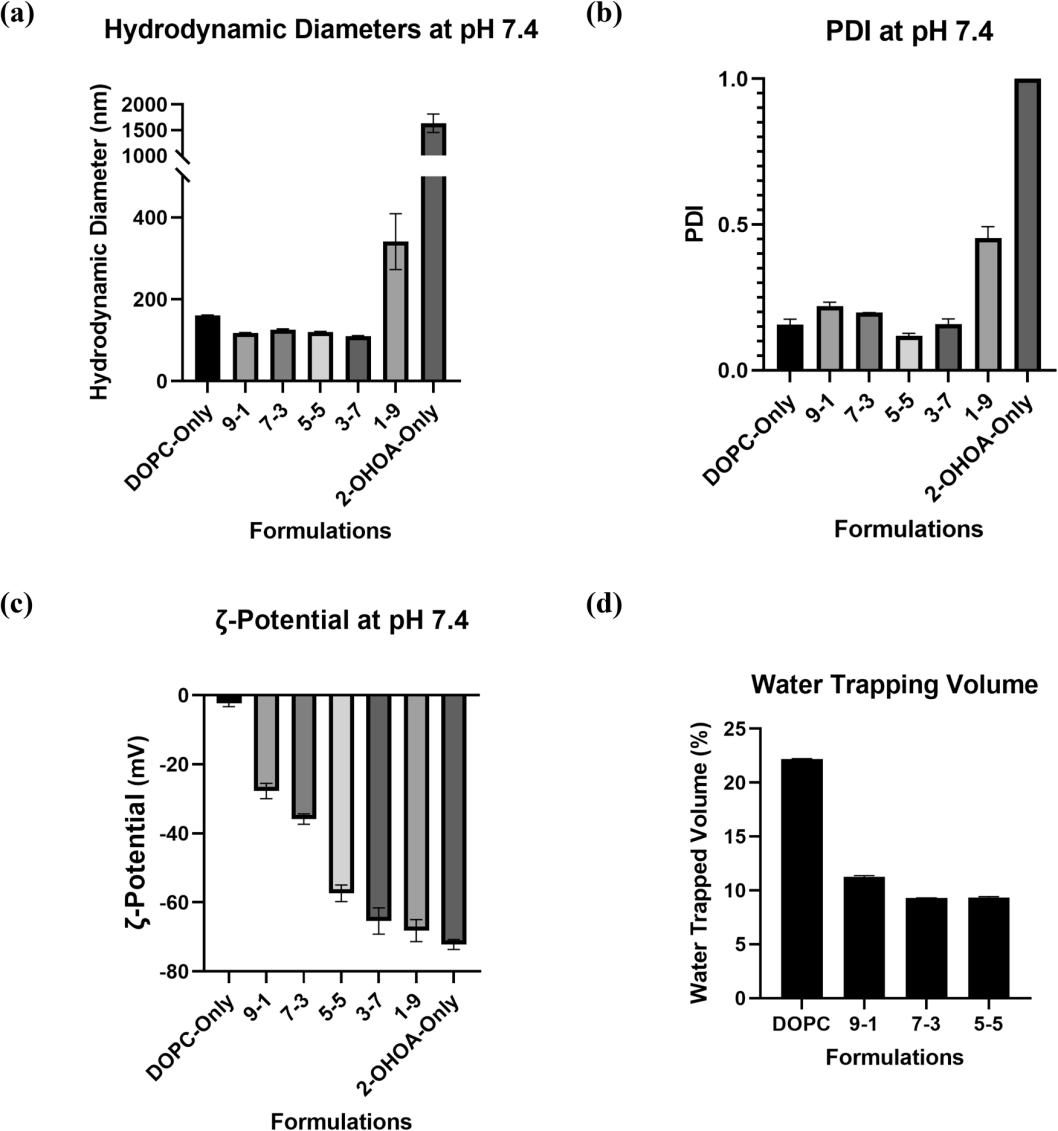
2-OHOA-embedded liposome characterization results. (**a**) Hydrodynamic diameter; (**b**) PDI and (**c**) ζ-potential. (**d**) Water trapping volume of 10 mM different liposomes. For DLS and ζ-potential investigation, all the liposome samples were diluted into 100 μM using D-PBS and the measurement were carried out at pH 7.4 and 25 °C, error bars represent ± *s.d*, *n* = 3.

After the liposome formulation, the DOPC amount was quantified using LabAssay Phospholipid kit (supplementary information Table S-1). The particle size, polydispersity index (PDI) and ζ-potential results are shown in Fig. 1a–c. Compared with DOPC-only liposomes, the presence of 2-OHOA in liposomes (9-1, 7-3, 5-5 and 3-7 formulations) slightly reduced mean hydrodynamic diameters. This phenomenon is attributed to the fact that, 2-OHOA incorporation decreases the packing order and increases the fluidity of liposomal membranes through intercalation between phosphorylcholine (PC) molecules; the kinked structure of the 2-OHOA hydrocarbon residue, akin to oleic acid, is believed to intensify this effect^13,21^. Increasing membrane fluidity can promote the formation of smaller liposomes^22^. In the absence of DOPC lipid, 2-OHOA formed particles with a hydrodynamic diameter at 1634.0 ± 180.4 nm and a PDI of 1, indicating that 2-OHOA alone could not form stable nano-sized particles in aqueous solution at a neutral pH.

Liposomes composed solely of DOPC exhibit ζ-potential value of − 2.40 ± 0.90 at a pH of 7.4. However, even low concentrations of incorporated 2-OHOA significantly reduced the ζ-potential of the liposomes. Moreover, ζ-potential of the 2-OHOA-embedded liposomes displayed a descending trend that aligns with the ratios of the 2-OHOA component. This is because of the carboxylic groups from 2-OHOA dissociate in aqueous solution, resulting in a negative surface charge^23^. Considering the structural stability of liposomes, the DOPC-only, 9-1, 7-3 and 5-5 liposome formulations were selected for the following research.

Water trapping efficiency is an important liposomal property in relation to the use of liposomes as drug carriers. The water trapping assay results (Fig. 1d) confirmed the existence of the inner aqueous phase of the prepared liposomes. Compared to the DOPC-only liposomes, the 2-OHOA-embedded liposomes showed a reduction of water trapping volume, which was attributed to the decreased particle size.

### Laurdan staining and investigation

#### Investigating the heterogenicity of cell membrane fluidity

In this study, the fluctuation in cancer cell membrane fluidity was chosen as the key parameter for assessing the MLT performance of the 2-OHOA-embedded liposomes. Different cells exhibit distinct membrane fluidity characteristics^24–26^. Using Laurdan *GP* quantification offers an efficient method for analyzing these fluidity variations. Cell membrane *GP* values can be influenced by factors, such as fluorescence acquisition settings and cell culture conditions^27,28^. To ensure consistency and establish a reliable baseline for cell status, all operations and measurement settings were consistently maintained.

**Figure 2:**
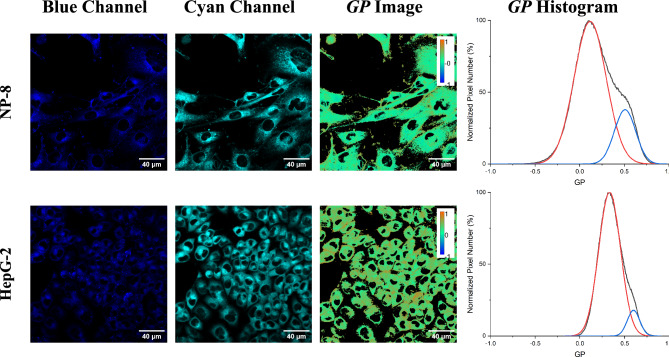
Two-photon microscopy Laurdan *GP* images and *GP* histograms of NP-8 (upper row) and HepG-2 (lower row) cells. From left to right, the first column displayed the Laurdan fluorescence images of blue channel (436/20 nm); the second column displayed the Laurdan fluorescence images of cyan channel (495/25 nm); the third columns display the pseudo-colored *GP* images; and, the fourth column displayed the pixel *GP* histograms obtained from the corresponding *GP* images. In the *GP* images, the orange represents maximum *GP* (1.0) and pure blue represents minimum *GP* (− 1.0). Scale bars represent 40 μm. In the *GP* histograms, the distribution of pixel-*GP* was deconvoluted by fitting two Gaussian distributions (blue and red lines) to the experimental data (black line).

As shown in Fig. 2, both HepG-2 and NP-8 cells exhibited heterogeneous *GP* patterns, consisting of ordered (higher *GP* values) and fluidic (lower *GP* values) membrane regions. The calculated average *GP* value of HepG-2 cell membrane was approximately 0.32, while the NP-8 cell membrane had an average *GP* value of approximately 0.2. A lower average *GP* value indicates a higher fluidity of the NP-8 cell membrane. Particularly, the high-*GP* regions are mainly distributed in the cell–cell contact regions and the cell margins, reflecting the condensed structure of the cell membranes^29^.

Laurdan fluorescence signals obtained from two-photon microscopy exhibit multiple pixel populations, corresponding to different membrane environments or lipid bilayer phases. To enable quantification and comparison, the pixel *GP* values derived from the *GP* images were normalized and presented in the form of histograms, referred to as *GP* histograms. The pixel count for a specific *GP* indicates the region of interest (ROI) associated with that particular population. The *GP* histogram of NP-8 cells (ranging approximately from − 0.5 to 0.8) displayed a broader distribution than that of HepG-2 cells (around − 0.1 to 0.8). This broader range of pixel *GP* values in NP-8 cells indicates greater diversity in cell membrane fluidity compared to HepG-2 cells.

Deconvolution helps to separate these pixel populations obtained from the *GP* images, providing a clearer understanding of the distinct lipid environments within the cellular membrane^29^. The *GP* histograms derived from the *GP* images were deconvoluted into two Gaussian distributions (Fig. 2). These distributions were categorized into a low-*GP* peak (illustrated by the red line), and a high-*GP* peak (illustrated by the blue line). The low-*GP* peaks are associated with the relatively fluidic membrane regions (shown as green in the *GP* images), and the high-*GP* peaks are associated with the more ordered membrane regions (shown as orange in the *GP* images). The deconvolution results are presented in Table S-2. Both NP-8 and HepG-2 cells exhibited a border distribution at low-*GP* peaks (compared according to the full width at half maximum, FWHM), indicating the presence of a predominantly fluidic membrane on the cells, as evidenced by the high-coverage area of the green pseudo-color regions on the *GP* images. The low-*GP* peaks of the NP-8 cell membrane were centered at 0.116 ± 0.015, whereas those of the HepG-2 cells were centered at 0.279 ± 0.030. However, concerning the high-*GP* peaks, NP-8 showed a center at 0.481 ± 0.24, and HepG-2 cells showed a center at 0.500 ± 0.058, with no apparent difference. Furthermore, there was no statistically significant difference in the high-*GP* region coverage (calculated according to the area under curve, AUC) between NP-8 (20.54 ± 6.98%) and HepG-2 (22.32 ± 7.20%). These findings imply that the difference in membrane fluidity between NP-8 and HepG-2 cells is mainly attributed to variations in the fluidic membrane (Ld) regions, rather than in ordered membrane (Lo) regions.

Laurdan two-photon microscopy revealed heterogeneous membrane fluidity among cell membranes, and distinct characteristics between NP-8 and HepG-2 cells. The higher membrane fluidity and heterogenicity observed in the NP-8 cell membrane can be attributed to a significantly lower sphingomyelin (SM) content in glioma cell membranes, as reported in previous studies^6,30^. SM interacts favorably with cholesterol and establishes the co-localization of SM and cholesterol in cell plasma membranes. The formed SM/cholesterol-rich domains are more ordered than the surrounding phase in biological membranes^31–33^. SM could also reduce the lateral heterogeneity in cholesterol-containing membranes. Specifically, unsaturated SM is able to accommodate both phosphorylcholine and cholesterol, forming a single phase, and maintaining membrane lipids in a homogeneous phase^34^. The reduced level of SM in glioma cells is considered be associated with its higher membrane fluidity and lateral heterogenicity.

#### Evaluating the influence of DOPC liposome on cell membrane fluidity

In this study, the liposome formulations utilized DOPC lipid. Thus, to minimize any additional impact on cell membranes, it is crucial to confirm the influence of DOPC lipid on cell membrane fluidity. HepG-2 and NP-8 cells were treated with varying concentrations of DOPC-only liposomes, and cell membrane fluidity was monitored over 48 h using Laurdan and a fluorescence spectrometer; the results are shown in Fig. S-1a. For concentrations up to 500 μM and a treatment duration of 48 h, DOPC-only liposomes did not induce significant changes in either HepG-2 or NP-8 cell membrane *GP* values. This observation is supported by two-photon microscopy images of Laurdan-stained cells (Fig. S-1b,c). These findings justified the exclusion of the influence of DOPC lipids on cell membrane fluidity. Thus, 2-OHOA was considered as the primary factor influencing cancer cell membrane fluidity in this study.

#### Investigating the impact of 2-OHOA-embedded liposome on cell membranes

**Figure 3:**
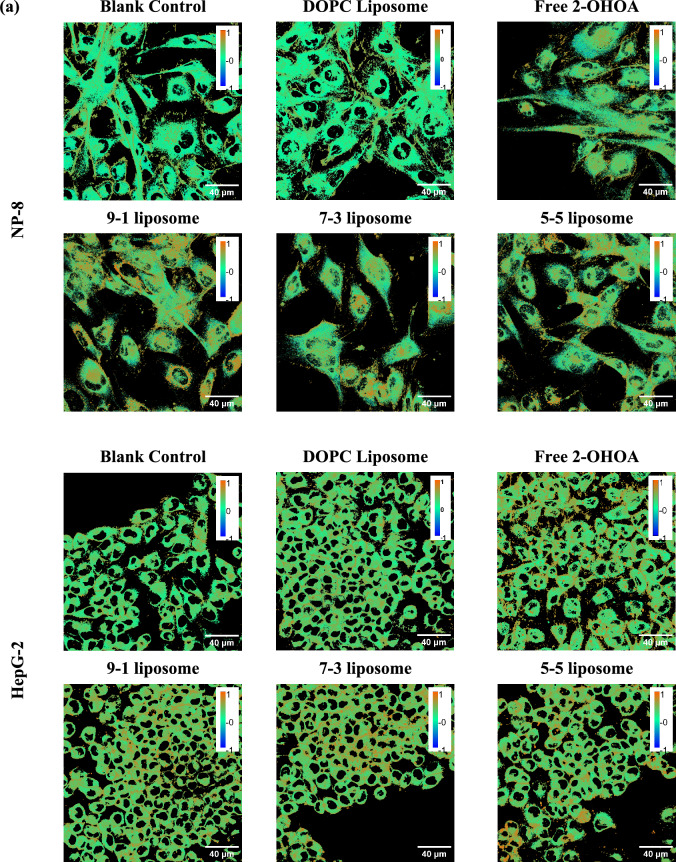

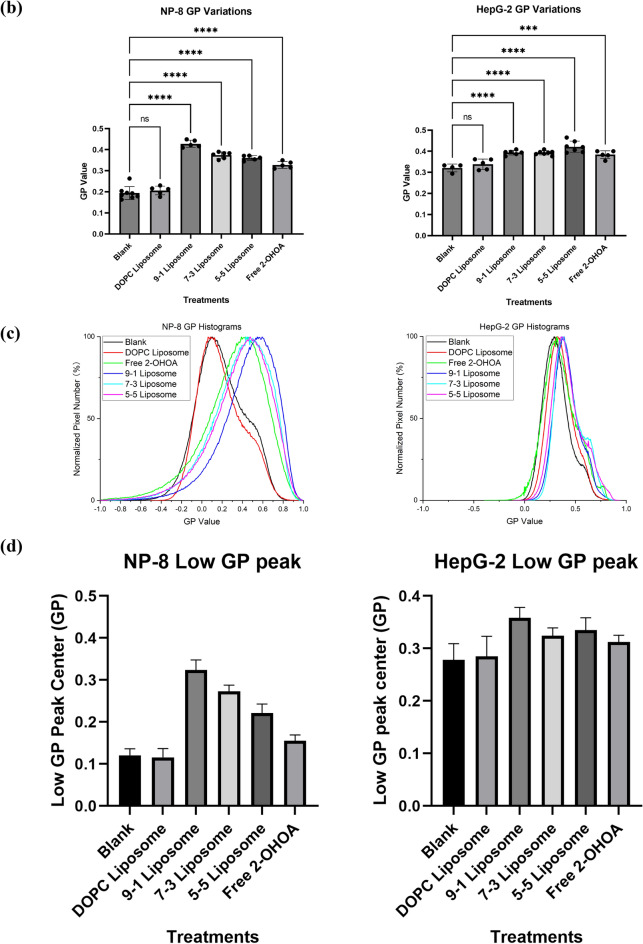
Laurdan two-photon microscopy imaging results: (**a**) *GP* images of NP-8 cells and HepG-2 cells with and without treatments. Scale bars represent 40 μm. (**b**) Average *GP* values calculated from the *GP* images. (**c**) *GP* histograms obtained from the *GP* images. (**d**) Summarized low-*GP* peak centers of deconvoluted results before and after treatment. In treatment groups, cells were incubated for 24 h with either 100 μM DOPC, 2-OHOA-embedded liposomes containing 100 μM 2-OHOA or 100 μM free 2-OHOA. Error bars represent ± *s.d*, (*n* = 4‒8). *ns*: no significant difference; ***: *p* < 0.001; ****: *p* < 0.0001.

Following a 24 h treatment with 2-OHOA-embedded liposomes (containing 100 μM 2-OHOA) or free 2-OHOA (100 μM), noticeable abundant high-*GP* regions (depicted in orange) emerged in both NP-8 and HepG-2 cell membranes, as shown in Fig. 3a. The calculated average *GP* values of cell membranes are summarized in Fig. 3b. Notably, NP-8 cells exhibited a more pronounced elevation in the average *GP* values than HepG-2 cells, indicating a more substantial impact of 2-OHOA on the NP-8 cell membrane. Specifically, for NP-8 cells, after a 24 h treatment with 2-OHOA, an increased SM concentration was observed (Fig. S-8). Analysis of normalized pixel *GP* histograms revealed distinct patterns of *GP* value elevation in the HepG-2 and NP-8 cell membranes. As shown in Fig. 3c, NP-8 cell *GP* histograms exhibited a pronounced rightward shift after treatments. Conversely, in the case of HepG-2 cells, the *GP* histograms showed a subtle rightward shift.

After deconvolving the *GP* histograms, a summary of the low-*GP* peak centers is provided in Fig. 3d. The low-*GP* peak shifting pattern reveals variations in high-fluidity membrane regions. After treatment, the NP-8 *GP* histograms exhibited a remarkable rightward shift. Additionally, as shown in Table S-2, the high-*GP* peak centers showed a slight rightward shift after treatment, accompanied by a significant increase in high-*GP* coverage (indicative of ordered membrane abundance). These findings suggest that, after 2-OHOA-embedded liposome treatment, NP-8 cells demonstrated a notable reduction in fluidity within the high-fluidity (Ld) membrane regions and an abundance of membrane areas exhibiting liquid-ordered (Lo) characteristics. For HepG-2, post 2-OHOA embedded liposome treatments, both low-*GP* peaks and high-*GP* peaks showed a subtle rightward shift. However, the high-*GP* coverage did not show a significant increase. The *GP* value variations of HepG-2 cell membranes were more moderate compared to NP-8 cells. Comparing the impacts of different liposome formulations, the 9-1 liposomes induced the most significant average *GP* increase (from ~ 0.2 to ~ 0.43) and the most pronounced shift in low-*GP* peak center (from ~ 0.12 to ~ 0.32) in NP-8 cells. Meanwhile, the 5-5 liposome induced a slightly higher average *GP* increase in HepG-2 cells compared to other formulations.

To assess the enhancement of liposome formulation, variations in the cell membrane Laurdan *GP* after formulated or non-formulated 2-OHOA treatments were investigated using a fluorescence spectrometer. The 9-1 liposome was chosen as the optimized formulation for NP-8 cells, and the 5-5 liposome was chosen as the optimized formulation for HepG-2 cells. Cells were incubated with varying concentrations of 2-OHOA-embedded liposomes or free 2-OHOA for 24 h, and the *GP* variation results are shown in Fig. S-2. Generally, the cell membrane *GP* elevation showed a 2-OHOA dose-dependent manner. Whereas the liposome formulations induced a more pronounced *GP* increase in both NP-8 and HepG-2 cells, this result is consistent with the two-photon microscopy observation results, reaffirming the enhancement of 2-OHOA performance after liposome formulation.

It is noteworthy that, an abundance of lipid droplets (LDs) in HepG-2 cells was observed after treatment with 2-OHOA-embedded liposomes or free 2-OHOA. The LDs were stained with Lipi-Red, a fluorescence probe designed for lipid droplet visualization, and observed by fluorescence microcopy (Fig. S-3a). After 2-OHOA treatment, a marked abundance of LDs was observed in HepG-2 cells. However, this phenomenon was not observed in NP-8 cells. A similar occurrence was reported in specific cell lines exposed to 2-OHOA^8,35^. This may be attributed to the structural similarity between 2-OHOA and oleic acid (OA), both of which belong to the monounsaturated omega-9 fatty acid category. An excess influx of OA into HepG-2 cells appears to trigger an interaction between LD and mitochondria, predominantly fostering LD growth^36^. 2-OHOA was found to induce a similar influence on HepG-2 cells. Notably, the lipid droplets attached on HepG-2 plasma membranes showed an extremely high *GP* value (0.5–0.8), contributing to the elevated *GP* values observed in HepG-2 cells after treatment (Fig. S-3b). This helps to explain the HepG-2 cells *GP* histograms showing an increase at *GP* ~ 0.6 after 2-OHOA treatment (Fig. 3c), which is attributed to the abundance of LDs.

In general, Laurdan two-photon microscopy serves as a powerful tool for visualizing the cell membrane fluidity variation. Treatment with 2-OHOA significantly increased the Laurdan *GP* values in both NP-8 and HepG-2 cells, with NP-8 cells exhibiting greater sensitivity. The distinct patterns in *GP* value elevation reveal the varied responses of NP-8 and HepG-2 cell to 2-OHOA treatments. The reduction in NP-8 cell membrane *GP* value is attributed to the overall decrease in plasma membrane fluidity, whereas HepG-2 cells exhibited reduction in a plasma membrane fluidity accompanied with an abundance of lipid droplets. Comparatively, the liposome formulation intensified the impact of 2-OHOA on NP-8 and HepG-2 cells, exhibiting promising enhancements in *GP* value alterations.

### LipiORDER staining and investigation

**Figure 4:**
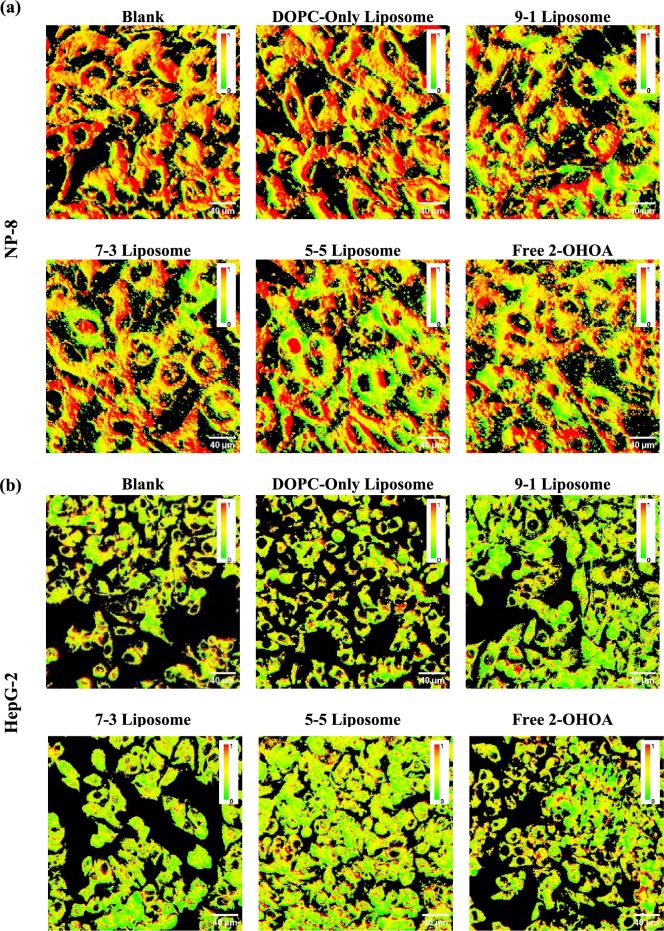

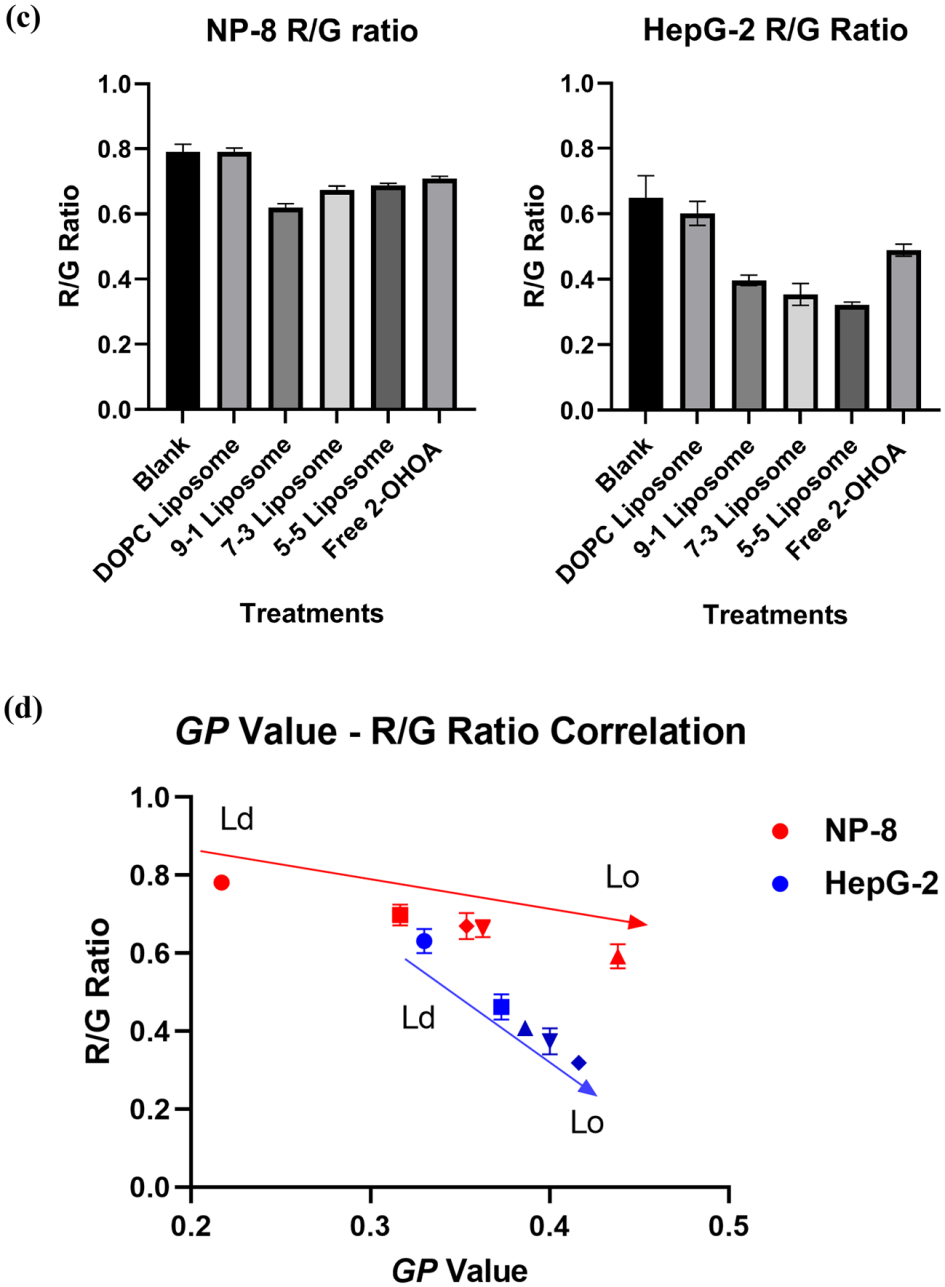
LipiORDER R/G ratio images of (**a**) NP-8 cells and (**b**) HepG-2 cells before and after treatments, green color represents Lo phase (low fluidity), and red color represents Ld phase (high fluidity), magnification is 40 times; Scar bar = 40 μm. (**c**) Summarized R/G ratio results of acquired images, error bars represent ± *s.d*, *n* = 5. (d) The *GP* value—R/G ratio correlations of NP-8 and HepG-2 cells with or without treatments. (●): Blank control; (■): freee-2-OHOA treated; (▲): 9-1 liposome treated; (▼): 7-3 liposome treated; (♦): 5-5 liposome treated. Error bars represent ± *s.d*, *n* = 3. In treatment groups, cells were incubated for 24 h with 100 μM DOPC, 2-OHOA-embedded liposomes containing 100 μM 2-OHOA or 100 μM free 2-OHOA.

To further validate the alterations in cell membrane lipid packing following treatment, we used another solvatochromic fluorescence probe, LipiORDER, to visualize changes in cell membrane lipid packing statues. When incorporated into the cell membrane, LipiORDER senses lipid packing and exhibit a fluorescent color shift, transitioning from green on liquid-ordered (Lo) membrane to red on liquid-disordered (Ld) membrane. Lipid packing in the cell membrane can be approximated and compared by quantifying the LipiORDER red/green fluorescence intensity (R/G) ratio. Figure 4a,b depicts the pseudo-colored R/G ratio images of NP-8 and HepG-2 cells before and after treatments. The average R/G ratios were summarized in Fig. 4c. Although both Laurdan *GP* value and LipiORDER R/G ratio provide insights into membrane properties, they offer complementary information rather than directly measuring the same aspect. Therefore, understanding both *GP* value and R/G ratio can provide a more comprehensive understanding of the membrane characteristics, including both fluidity and lipid packing order. Correlation analysis between cell *GP* value and R/G ratio variations before and after 2-OHOA treatments (summarized in Fig. 4d) revealed a linear relationship, solidifying the proportional relation between cell membrane fluidity/polarity and cell membrane lipid packing.

Initially, both HepG-2 and NP-8 cells displayed heterogeneous plasma membrane lipid packing (Fig. 4a,b), comprising Lo phase (green) and Ld phase (red). NP-8 cells generally exhibited a more disordered membrane lipid packing than HepG-2 cells. Treatment with DOPC-only liposomes did not induce noticeable changes in average R/G ratios. While, after 2-OHOA treatments (formulated or non-formulated), significant R/G ratio decreases were observed in both HepG-2 and NP-8 cells, indicating an increase in membrane lipid packing. Also, NP-8 and HepG-2 cells exhibited distinct R/G ratio variations after 2-OHOA treatments. NP-8 cells showed an abundance of Lo phase on the cell plasma membrane after 2-OHOA treatments (Fig. 4a). Whereas HepG-2 cells showed a reduced R/G ratio in plasma membrane, accompanied with a marked green colored region in cytoplasm (Fig. 4b), which is associated with the abundant lipid droplets stained by LipiORDER. As mentioned previously, 2-OHOA treatment induced significant abundance of LDs in HepG-2 cells, but not observed in NP-8 cells. The LDs exhibit intense green fluorescence when staining with LipiORDER^19,37^. Notably, in comparison to two-photon microscopy, fluorescence microscopy captures a thicker section, leading to the detection of more cytoplasmic LDs and resulting in a pronounced decrease in the average R/G ratio. Furthermore, analysis of the R/G ratio across various groups (Fig. 4c) reveled that, compared to the non-formulated 2-OHOA treatment, the liposome formulated 2-OHOA induced more significant R/G ratio decrease. Specifically, 9-1 liposomes induced the most pronounced R/G ratio decrease in NP-8 cells and the 5-5 liposomes induced most significant R/G ratio decrease in HepG-2 cells. This trending is closely aligning with the Laurdan *GP* investigation results, solidified that, the liposome formulation significantly enhanced the impact of 2-OHOA on cancer cells.

In summary, the LipiORDER R/G ratio results are consistent with the Laurdan *GP* value results. Treatment with 2-OHOA-embedded liposomes, as well as free 2-OHOA, notably improved the packing of cancer cell membrane lipids, effectively reducing cell membrane fluidity. However, distinct patterns of R/G ratio variation were also observed in NP-8 and HepG-2 cells. The abundance of LDs induced by 2-OHOA treatment significantly contributed to a decrease in R/G ratio in HepG-2 cells. Furthermore, different liposome formulations resulted in varying extents of R/G ratio decrease in both NP-8 and HepG-2 cells. These findings underscore the impact of 2-OHOA on cancer cell membrane lipid packing, while also suggesting that different liposome formulations, despite containing the same amount of 2-OHOA, exhibited diverse effects on cancer cell membrane properties. In the subsequent study, we will investigate the factors that influence the performance differences among various 2-OHOA-embedded liposome formulations.

### Cellular internalization efficacy and endocytic mechanism

Considering the diverse effects induced by various liposome formulations on cell membrane fluidities, we hypothesize that differences in cell internalization efficacy and cellular uptake mechanisms are likely contributors to variations in 2-OHOA performance (as illustrated in Fig. S-4). The efficiency of cellular internalization and endocytic mechanisms of nanoparticles (NPs) are dependent on various factors, including the physicochemical and surface properties of NPs^38,39^. Importantly, different cell types may utilize distinct endocytic pathways for internalization of the same NPs^40,41^. To improve the drug delivery efficacy of 2-OHOA, it is crucial to evaluate both cellular internalization efficiency and the endocytic mechanism.

**Figure 5:**
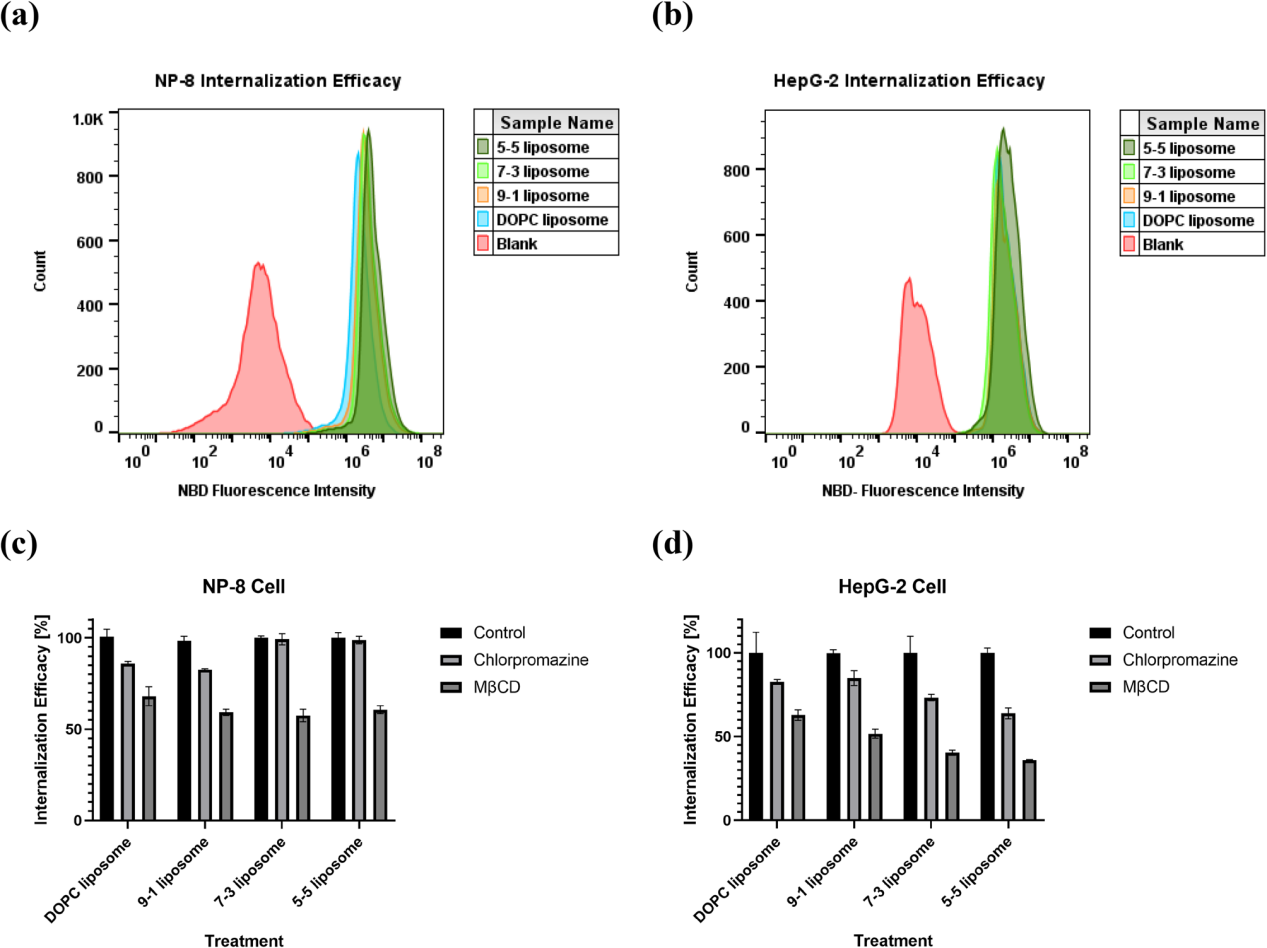
Liposome internalization characterization results. (**a**,**b**) Histograms of NP-8 and HepG-2 cellular internalization efficacy; (**c**,**d**) NP-8 and HepG-2 internalization efficacy with and without endocytosis inhibitions. Error bars represent ± *s.d* (*n* = 3).

The results of cellular internalization efficiency are presented in Fig. 5a,b. After a 6-h incubation, liposomes of different formulations did not exhibit significant differences in cellular internalization efficiency in NP-8 cells. However, for HepG-2 cells, the 5-5 liposome showed a slightly enhance internalization efficacy, potentially contributing to a slightly higher impact of the 5-5 liposome on HepG-2 cells. To assess the endocytic mechanism of 2-OHOA-embedded liposomes, methyl-β-cyclodextrin (MβCD) was utilized to inhibit the caveolin-mediated endocytosis pathway, and chlorpromazine was used to inhibit the clathrin-mediated endocytosis pathway^42^. As shown in Fig. 5c,d, the cellular endocytic pathway of liposomes varied depending on the cell type and liposome formulation. In NP-8 cells, endocytosis of these liposomes was primarily caveolae-dependent. Inhibition of clathrin reduced the internalization of DOPC-only and 9-1 liposomes, but it did not affect the uptake of 7-3 liposomes and 5-5 liposomes, suggesting that 7-3 liposomes and 5-5 liposomes are not internalized by NP-8 cells via the clathrin-dependent endocytosis pathway. In HepG-2 cells, both DOPC-only liposomes and 9-1 liposomes displayed a similar clathrin-dependent endocytosis pathway. Moreover, with an increase in the ratio of the 2-OHOA compound, the liposomes exhibited heightened endocytosis-dependent internalization by HepG-2 cells.

The summarized results, including liposome endocytic ratios and cell membrane *GP* variations, are presented in Table 1. Interestingly, considering the previously mentioned variations in cell membrane fluidity after treatment, the 9-1 liposomes induced a more pronounced membrane fluidity reduction in NP-8 cells than the 7-3 and 5-5 liposomes. Meanwhile, the 5-5 liposomes exhibited an enhanced impact on HepG-2 cells. Notably, cell *GP* variations were heightened with an increase in liposome endocytic ratio. These outcomes indicate that the endocytosis of 2-OHOA-embedded liposomes intensifies the influence of 2-OHOA on cancer cells, leading to a more significant reduction in cell membrane fluidity. Typically, in the process of caveolae-mediated endocytosis, nanoparticles do not fuse with lysosomes after their entry into cells; via this endocytic pathway, the drug payload can be delivered to endoplasmic reticulum (ER) or Golgi apparatus, increasing the accumulation of drugs in ER or Golgi apparatus^43,44^. 2-OHOA has been reported as a sphingomyelin synthase (SMS) activator. SMS has two subtypes: SMS1 and SMS2. Partial SMS2 is localized to the plasma membrane, whereas most SMSs are localized in the inner plasma trans-Golgi network (TGN) rather than in the cytoplasm^45^. The endocytosis pathway associated with 2-OHOA-embedded liposomes is believed to promote interactions between 2-OHOA and SMS, leading to enhanced SM abundance, ultimately reduce the cell membrane fluidity (*GP* increase).

**Table 1:** Cell endocytosis ratio and *GP* variations (*n* = 3).

Cell line	Liposome formulations	Clathrin-mediated endocytosis ratio (%)	Caveolin-mediated endocytosis ratio (%)	*GP* variation (%)
NP-8	9-1	17.3 ± 0.4	40.8 ± 1.69	95.6 ± 2.1
	7-3	0.8 ± 3.1	42.6 ± 3.5	85.6 ± 5.1
	5-5	1.3 ± 2.0	39.3 ± 2.1	65.7 ± 6.1
HepG-2	9-1	15.1 ± 4.4	48.2 ± 2.7	22.7 ± 3.6
	7-3	26.7 ± 1.9	59.7 ± 1.5	23.1 ± 1.6
	5-5	36.1 ± 3.3	64.2 ± 0.4	35.6 ± 4.7

These results support the hypothesis that the varied effects of different liposome formulations on cancer cells are associated with diverse cellular uptake mechanisms. The endocytic uptake pathway is believed to amplify the influence of 2-OHOA-embedded liposomes on cancer cells.

### Anticancer performance of 2-OHOA-embedded liposomes

Based on previous results, the 9-1 liposome induced more dramatic membrane fluidity changes in NP-8 cells than free 2-OHOA. While the 5-5 liposomes induced greater membrane fluidity changes in HepG-2 cells. Moreover, analysis via the MTT assay on normal cells (OUMS-36T cell line) demonstrated that various liposome formulations exhibited comparable and modest inhibition on OUMS-36T at different concentrations (Fig. S-5), indicating low cytotoxicity of 2-OHOA on healthy cells. To evaluate the anticancer performance enhancement of the liposome formulation on cancer cells, an apoptosis assay was conducted and the results are shown in Fig. S-6. Compared to non-formulated 2-OHOA, the 9-1 liposome induced a higher cellular apoptotic ratio, particularly the late apoptotic ratio in NP-8 cells, suggesting superior anticancer efficacy of the liposome formulations. Similar results were observed in HepG-2 cells.

Notably, previous studies have reported augmentations in the fluidity of various cell membranes during apoptosis induced by specific agents^46–48^. During apoptosis process, cells undergo a loss of membrane lipid asymmetry. SM, predominantly abundant in the outer leaflet of the cell membrane, is transferred to the inner leaflet, leading to a depletion of the Lo phase^49^. However, in this study, we did not observe an elevation in cancer cell membrane fluidity following 2-OHOA treatment, despite the induction of apoptosis in these cells. The prevalence of the Lo phase on the cancer cell membrane following 2-OHOA treatment is presumed to overshadow the anticipated increase in fluidity during apoptosis.

From our data, it is evident that the liposome formulation not only enhanced the impact of 2-OHOA on cancer cell membrane fluidity but also augmented the anticancer performance of 2-OHOA compared to non-formulated 2-OHOA.

## Conclusion

Using Laurdan two-photon microscopy, we demonstrated that both 2-OHOA and 2-OHOA-embedded liposomes effectively reduced the fluidity of NP-8 and HepG-2 cell membranes. The liposome formulation generally intensified the impact of 2-OHOA on both NP-8 and HepG-2 cell membrane fluidity, although distinct patterns were observed in the descending membrane fluidity. LipiORDER fluorescence microscopy investigation further validated the variations in cell membrane lipid packing after 2-OHOA-embedded liposome treatment. Our investigation was extended to exploring cellular internalization efficacy and endocytic mechanisms, revealing an endocytosis-dependent enhancement of 2-OHOA-embedded liposome performance. Additionally, the enhanced anticancer performance of the liposome formulation was validated.

These findings substantiate the hypothesis that the liposome formulation not only addresses the solubility challenges of 2-OHOA, but also enhances its therapeutic efficacy as a membrane lipid therapy drug. Future research endeavors should explore a range of lipid-based nano-drug delivery systems to identify optimal formulations for delivering 2-OHOA. To advance these findings, it would be beneficial to conduct tests in animal models, focusing specifically on understanding the endocytic pathways of nanoparticles.

## Materials and methods

### Materials

1,2-dioleoyl-*sn*-glycero-3-phosphocholine (DOPC), 2-hydroxyoleic acid (2-OHOA), sphingomyelin (Brain, Porcine) and 1,2-dipalmitoyl-*sn*-glycero-3-phosphoethanolamine-*N*-(7-nitro-2-1,3-benzoxadiazol-4-yl) (16:0 NBD PE) were purchased from Avanti Polar Lipids. 6-Dodecanoyl-2-Dimethylaminonaphthalene (Laurdan) was purchased from Thermo Fisher Scientific. LipiORDER was purchased from Funakoshi. BD Pharmingen™ FITC Annexin V Apoptosis Detection Kit I was purchased from BD Biosciences. Calcein was purchased from TCI Chemicals. 3-(*N*-Morpholino) propanesulfonic Acid (MOPS free acid), Sodium acetate, Ethylenediamine-*N*, *N*, *N′*, *N′*-tetraacetic acid (EDTA-2Na), and Lipi-Red was purchased from Dojindo Laboratories. Chloroform, Dimethyl sulfoxide (DMSO), Fetal bovine serum (FBS), Eagle’s Minimum Essential Medium (E-MEM), Dulbecco’s Modified Eagle Medium (D-MEM), Trypsin (0.25 w/v%, EDTA solution with Phenol Red), LabAssay Phospholipid kit, MTT (3-(4,5-Dimethylthiazol-2-yl)-2,5-Diphenyltetrazolium Bromide), Penicillin–Streptomycin solution, Anhydrous Cobalt (II) Chloride (CoCl_2_) and D-PBS were purchased from Fujifilm Wako Pure Chemical.

### Preparation and characterization of liposomes

#### Liposomes preparation

Liposomes were prepared using the thin-film hydration-extrusion method. In brief, DOPC and 2-OHOA were first dissolved in chloroform and mixed in varying ratios in 100 mL round-bottom flasks. These lipid solutions underwent vacuum evaporation at 60 °C, followed by maintenance under high vacuum conditions at room temperature for 24 h. Following the vacuum step, D-PBS was added to the flasks to hydrate lipid films. The resulting vesicle suspensions were subjected to 4 cycles of freezing at − 80 °C and thawing at 65 °C. After the freeze–thaw process, the suspensions were extruded 13 times through a polycarbonate membrane with an average pore diameter of 200 nm, using an extruder (LiposoFast LF-1, Avestin, Canada).

The DOPC:2-OHOA ratios were 10:0, 9:1, 7:3, 5:5, 3:7, 1:9, and 0:10 (molar ratios). For brevity, liposomes fabricated from different formulations and 2-OHOA-only particles were named as follows: DOPC-only liposomes, 9-1 liposomes, 7-3 liposomes, 5-5 liposomes, 3-7 liposomes, 1-9 liposomes, and 2-OHOA-only particles. To investigate the cellular internalization efficiency and uptake mechanism, 1 mol% 16:0 NBD-PE was incorporated into the liposome formulations before the vacuum-evaporation step.

#### Particle size & ζ-potential characterization

Hydrodynamic particle size, polydispersity index (PDI), and ζ-potential were investigated using a Zetasizer (ZEN5600, Malvern, UK). Various liposome suspensions were diluted to a concentration of 100 μM using D-PBS. These measurements were conducted in triplicate at a temperature of 25 °C.

#### Evaluation of trapped water volume in liposomes

The trapped water volume in liposomes was investigated using the calcein-CoCl_2_ quenching method, following a previously reported method with slight modifications^50^. Briefly, after the preparation of lipid thin films in round bottom flasks, the lipid thin films were hydrated using MOPS buffer (20 mM MOPS free acid, 5 mM sodium acetate, and 1 mM EDTA) containing 10 μM calcein. When the calcein concentration was lower than 20 μM, a linear concentration-fluorescence intensity relation was observed. The liposome preparation procedure was consistent with that previously described. The prepared liposomes containing calcein were diluted for 10 times before fluorescence spectrometer measurements. The calcein fluorescence intensity (FI) in the suspension was measured using a fluorescence spectrometer (FP-8500, Jasco, Japan), with excitation at 495 nm and emission at 515 nm. The total calcein FI was measured and named as ${FI}_{total}$. After addition of CoCl_2_ solution at a final concentration of 0.25 mM, the calcein in outer aqueous phase was quenched, then FI was measured and namely ${FI}_{in}$. Then 10 (v/v) % Triton X-100 solution was added into the sample at a final concentration of 1 (v/v) % to disrupt the liposomes structure and the FI was measured and referred to as ${FI}_{TX}$, which is the FI of CoCl_2_-calcein equilibrium statues. The dilution factors (DF) were calculated using volume ratios. Water trapped volume (% of total volume) was calculated using Eq. (1).

1 $$Water\; trapped \;volume \left(\% \;of \;total \;volume\right)=\frac{{{FI}_{in}\times {DF}_{1}-FI}_{TX}\times {DF}_{2}}{{FI}_{total}{-FI}_{TX}\times {DF}_{2}}\times 100 \%$$

In this equation, ${DF}_{1}$ is the dilution factor after addition of CoCl_2_ solution, ${DF}_{2}$ is the dilution factor after addition of Triton X-100 solution.

### Cell culture and treatment

#### Cell culture

HepG-2, NP-8, and OUMS-36T cells obtained from the Japanese Collection of Research Bioresources (JCRB) were used in this study. HepG-2 was cultured in E-MEM media; NP-8 and, OUMS-36T cells were cultured in D-MEM media. All cell culture media were supplemented with 10% v/v FBS and streptomycin-penicillin. Cells were cultured at 37 °C in in a humidified atmosphere containing 5% CO_2_.

#### MTT assay

Cell viability was evaluated using the MTT assay. Briefly, OUMS-36T cells were seeded in 96-well plates at a concentration of 5000 cells/well. After 48 h incubation, the cells were washed with D-PBS before adding fresh medium containing different liposomes. The liposome concentrations were set at 100, 200, 300, 400, and 500 μM (calculated based on the total lipid amount). Following 24 and 48 h of incubation, the cells were washed again and incubated for 4 h with fresh medium containing 0.5 mg/mL MTT. After incubation, the 96-well plates were centrifuged at 1000 × g for 5 min, and the media was carefully aspirated. Subsequently, DMSO was added to the wells, followed by a 30-min incubation to dissolve the formazan crystals. The optical densities (OD) of the resulting solutions were measured at 570 nm using a spectrophotometer (xMark™ Microplate Absorbance Spectrophotometer, Bio-Rad, USA). The cell viability was calculated at OD_570 nm_.

#### Cellular internalization assay and endocytosis inhibition

To investigate the cellular internalization efficacy of different liposomes, each liposome formulation was doped with 1 mol % of 16:0 NBD-PE during the preparation procedure. The cells were exposed to 100 μM 16:0 NBD-PE labeled liposomes 6 h. After treatment, the cells were thoroughly washed and collected for analysis using flow cytometer (Applied Biosystems Acoustic focusing cytometer, Attune, USA).

To confirm the endocytic mechanism of liposomes, endocytosis was inhibited using 10 μg/mL chlorpromazine, an inhibitor of clathrin-mediated endocytosis; or 2.0 mM methyl-β-cyclodextrin (MβCD), an inhibitor of caveolae-mediated endocytosis. Each endocytosis inhibitor was added to culture medium for 30 min before the addition of 16:0 NBD-PE labeled liposomes.

#### Apoptosis assay

NP-8 and HepG-2 cells were treated with 2-OHOA-embedded liposome (containing 100 μM 2-OHOA) or non-formulated 2-OHOA (100 μM) for 48 h. The apoptosis rate was assessed using FITC Annexin V Apoptosis Detection Kit (BD Pharmingen™, BD Biosciences, USA) according to the manufacturer’s instructions.

### Cell staining and investigation

#### Laurdan staining and investigation

Laurdan was dissolved in DMSO at a concentration of 1 mM as a stock solution. To measure the steady-state Laurdan fluorescence spectrum in cell membrane, HepG-2 and NP-8 cells were seeded in 6-well plates. After 24 h treatments with different liposomes or free 2-OHOA (firstly dissolved in DMSO at a concentration of 20 mM as stock solution, then diluted in cell culture media for cell treatment), the culture media was carefully removed, and the cells were gently washed with D-PBS. Subsequently, fresh pre-heated media containing 10 μM Laurdan was introduced into the wells and incubated for 30 min in a cell culture incubator shielded from light. Following Laurdan staining, cells were washed and detached using trypsin. The collected cells were suspended in D-PBS and analyzed using a fluorescence spectrometer (FP-8500, Jasco, Japan). Steady-state Laurdan spectra were obtained with an excitation wavelength of 345 nm, and emission was collected in the range of 400–600 nm. In the blank control group, cells from 3 replicate wells were stained with Laurdan and analyzed. For each treatment group (including each liposome formulation and free 2-OHOA treatment), cells from 5 replicate wells were stained with Laurdan and analyzed. Each replicate of cell samples was measured 3 times, and the Laurdan spectra were averaged across the 3 measurements.

The Laurdan steady-state fluorescence spectra data from fluorescence spectrometer were collected and analyzed. The ${GP}_{s}$ value (*GP* value calculated according to steady-state Laurdan spectra) was calculated according to the Eq. (2):

2 $${GP}_{s}=\frac{({I}_{440}-{I}_{490})}{({I}_{440}+{I}_{490})}$$

where ${I}_{440}$ and ${I}_{490}$ represent the fluorescence intensity at 440 and 490 nm, respectively.

For two-photon microscopy observations, cells were initially cultured in 35 mm Φ glass-bottom dishes. Laurdan staining was performed as previously described. Following staining, the samples were observed under a two-photon microscopy. To maintain the temperature and CO_2_ concentration of the cell samples during microscopy observation, the glass bottom dishes were placed in a living cell imaging chamber equipped with a stage-top incubator (INUB-PPZI, Tokai Hit, Japan) to sustain a 37 °C and 5% CO_2_ environment. Two-photon fluorescence images of the Laurdan-labeled cells were obtained with an inverted microscopes (Eclipse TE2000-U, Nikon, Japan) with a × 60 water-immersion objective (Plan Apo VC 60 × , NA = 1.2, Nikon, Japan). A Ti–sapphire laser (Chameleon Vision II, Coherent, USA) with a repetition rate of 80 MHz and pulse width of 140 femtosecond (fs) was used as the excitation laser. The wavelength peak was tuned to 780 nm and the power was adjusted to 100 mW. The group delay dispersion (GDD) was adjusted to 14,000 femtosecond squared (fs^2^). Laurdan emission from the cell samples were filtered through 436/20 nm (blue) and 495/25 nm (cyan) bandpass filters. The fluorescence intensity of two channels were detected using a laser-scanning fluorescence detector (D-Eclipse C1, Nikon, Japan). The relative sensitivities of the two channels were determined using 100 μM Laurdan in DMSO (spectrum shown in Fig. S-7), and the calibration factor (G-factor) was calculated (refer to supplementary information). Two-photon microscopy images of Laurdan-stained cell membrane were analyzed using the imageJ software (ImageJ 1.53t. https://imagej.net/ij/). Laurdan *GP* images were acquired by calculating the *GP* value of each pixel. The ${GP}_{m}$ (*GP* value calculated according to Laurdan two-photon microscopy images) of each pixel, was calculated according Eq. (3).

3 $${GP}_{m}=\frac{{I}_{blue}-({G}_{Laurdan}\times {I}_{cyan})}{{I}_{blue}+({G}_{Laurdan}\times {I}_{cyan})}$$

In this equation, ${I}_{blue}$ is the fluorescence intensity of the blue channel and ${I}_{cyan}$ is the fluorescence intensity of the cyan channel; ${G}_{Laurdan}$ is the Laurdan calibration factor (G factor). The *GP* values of pixels were obtained using image J software and the *GP* histograms were deconvoluted using Origin software (Origin 2023 v.10.0. https://www.originlab.com/). For blank control group and each treatment group (including each liposome formulation and free 2-OHOA), 3 replicate plates of cell samples were stained with Laurdan and imaged. 3 to 5 images were obtained from each plate of cells, with each image generated by averaging 4 scanning frames.

#### LipiORDER staining and imaging

LipiORDER was dissolved in DMSO at a concentration of 10 μM as a stock solution. Cells cultured in 35 mm Φ glass-bottom dishes were carefully washed with D-PBS, followed by incubation with 300 nM of LipiORDER in D-PBS for 15 min. After the staining period, the cells were rinsed with D-PBS and subsequently examined using a fluorescence microscopy (BX53, Olympus, Japan) equipped with image-splitting optics (W-View Gemini A12801-01, Hamamatsu, Japan). For excitation, a light filter with a wavelength of 388/38 nm was used. The emitted light was directed through a dichroic mirror, separating it into green channel (510/84 nm) and red channel (> 570 nm). Images from both the green and red channels were captured with an exposure time of 200 ms. The ratiometric analysis of LipiORDER fluorescence images was performed using the ImageJ software (ImageJ 1.53t. https://imagej.net/ij/). The backgrounds of the green and red channel images were first subtracted, and the R/G ratio images were acquired by calculating the fluorescence intensity ratio between red channel image and green channel image according to Eq. (4).

4 $$R/G\; ratio=\frac{{I}_{Red}}{{I}_{Green}}$$

In this equation, the ${I}_{Red}$ is the LipiORDER fluorescence intensity from red channel, the ${I}_{Green}$ is the LipiORDER fluorescence intensity from green channel. For blank control group and each treatment group (including each liposome formulation and free 2-OHOA treatment), 3 plates of cell samples were stained with Laurdan for fluorescence microscopy imaging. 3 to 5 images were obtained from each plate of cells (exposure time of 200 ms).

#### Lipid droplets staining and imaging

Lipi-Red was dissolved in DMSO at a concentration of 1 mM and used as a stock solution. Cells cultured in glass bottom dishes were stained with Lipi-Red probe, according to the manufacturer’s instructions. After staining, the cells were rinsed carefully with D-PBS and observed using fluorescence microscopy (BX53, Olympus, Japan) at an excitation wavelength of 530–550 nm and the emission fluorescence signal was detected at wavelength > 575 nm.

#### Cell membrane sphingomyelin quantification

Sphingomyelin from cell membranes was quantified using a high-performance thin-layer chromatography (HPTLC)-densitometry method. Following the treatment, the cell membrane lipids were extracted using the Folch method. Phospholipids extracted from the cell membrane were quantified using the LabAssay Phospholipid Kit. Lipids were separated via HPTLC on Whatman silica gel-60 plates, using a mobile phase consisting of chloroform/ethanol/water/tri-ethylamine in a volume ratio of 30:35:7:35. Following HPTLC separation, the plates were air-dried, sprayed with a solution containing 8% (wt/vol) H_3_PO_4_ and 10% (wt/vol) CuSO_4_, and then charred at 180 °C for 10 min. Subsequently, the plates were imaged using a ChemiDoc system, and sphingomyelin (SM) amount was compared based on the staining density.

### Supplementary Information

Supplementary Information.

## Data Availability

Data will be available upon request. The corresponding authors should be contacted for any data required for the conducted study.
