# Supplementary material for: Investigating the impact of 2-OHOA-embedded liposomes on biophysical properties of cancer cell membranes via Laurdan two-photon microscopy imaging
Source: Sci Rep. 2024 Jul 9;14:15831. doi: 10.1038/s41598-024-65812-9 (PMC11233574; doi:10.1038/s41598-024-65812-9)
Supplement: Supplementary file 1 — Supplementary Information. [file 41598_2024_65812_MOESM1_ESM.docx]

**Supplementary Information**

**Investigating the impact of 2-OHOA-embedded liposomes on biophysical properties of cancer cell membranes via Laurdan two-photon microscopy imaging**

Xuehui Rui^1,^ **^*^**, Yukihiro Okamoto^1^, Shuichiro Fukushima^2^, Nozomi Morishita Watanabe^1^, and Hiroshi Umakoshi^1,^ **^*^**

1. Division of Chemical Engineering, Graduate School of Engineering Science, Osaka University, 1-3 Machikaneyamacho, Toyonaka, Osaka 560-8531, Japan.
2. Department of Mechanical Science and Bioengineering, Graduate School of Engineering Science, Osaka University, 1-3 Machikaneyamacho, Toyonaka, Osaka 560-8531, Japan.

**Corresponding Authors E-mails:**

**^*^**Hiroshi Umakoshi: [umakoshi.hiroshi.es@osaka-u.ac.jp](mailto:umakoshi.hiroshi.es@osaka-u.ac.jp)

**^*^**Xuehui Rui: [xuehui.rui@cheng.es.osaka-u.ac.jp](mailto:xuehui.rui@cheng.es.osaka-u.ac.jp)

**Investigating the impact of 2-OHOA-embedded Liposomes on biophysical properties of cancer cell membranes via Laurdan two-photon microscopy imaging**

This supplementary information contains the following sections:

1. Liposome DOPC quantification results;
2. Influence of DOPC on cell membrane fluidities investigation results;
3. *GP* histograms deconvolution results;
4. Comparison of the influence of 2-OHOA-embedded liposomes and free 2-OHOA (fluorescence spectrometer measurements);
5. Lipid droplet staining and observation;
6. Illustration of endocytosis-dependent enhancement of 2-OHOA liposome performance;
7. MTT assay results;
8. Anticancer performance of 2-OHOA embedded liposome;
9. G-factor calibration of *GP* images using a reference solution;
10. Sphingomyelin high-performance thin film chromatography (HPTLC) results
11. **DOPC Quantification**

**Table. S-1 DOPC quantification result (*n*=3)**

| **Formulation** | **DOPC-only** | **9-1** | **7-3** | **5-5** | **3-7** | **1-9** |
| --- | --- | --- | --- | --- | --- | --- |
| **DOPC concentration (mg/mL)** | 3.75 ± 0.16 | 3.57 ± 0.40 | 2.79 ± 0.08 | 2.16 ± 0.04 | 1.15 ± 0.03 | 0.42 ± 0.01 |
| **DOPC concentration (mM)** | 5.11 ± 0.21 | 4.86 ± 0.55 | 3.81 ± 0.11 | 2.94 ± 0.06 | 1.56 ± 0.04 | 0.57 ± 0.01 |
| **Total concentration (mM)** | 5.11 ± 0.21 | 5.40 ± 0.61 | 5.44 ± 0.14 | 5.88 ± 0.11 | 5.21 ± 0.13 | 5.66 ± 0.06 |

Total concentration refers to the combined sum of DOPC and 2-OHOA concentrations. LabAssay Phospholipid kit was used to quantify the DOPC in liposomes according to the manual.

1. **Influence of DOPC on cell membrane fluidities**

| **(a)** | **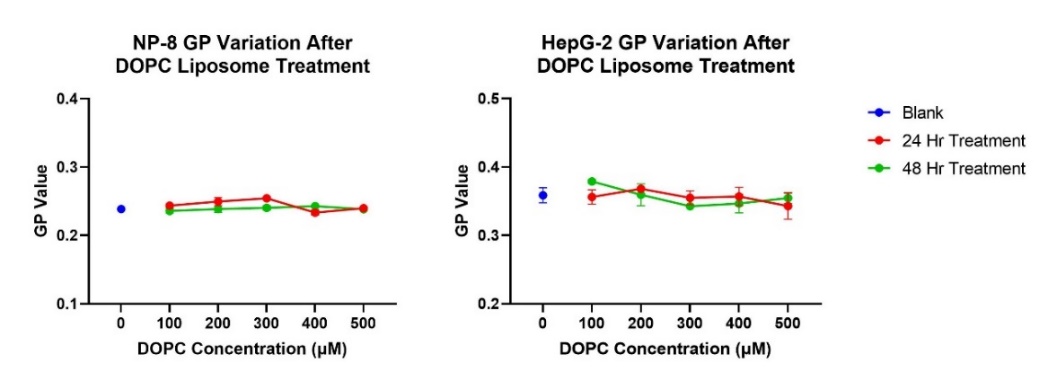** | |
| --- | --- | --- |
| **(b)** | **NP-8 Cells** | |
| **Blank** | **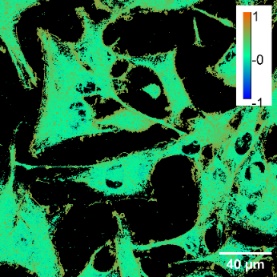** | **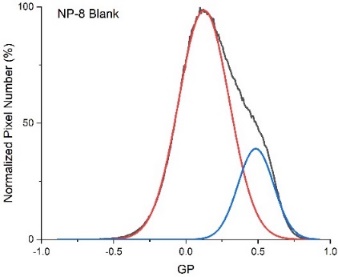** |
| **DOPC Treated** | **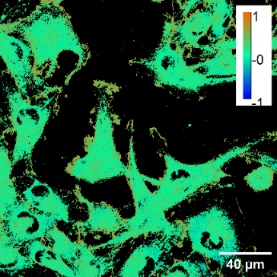** | **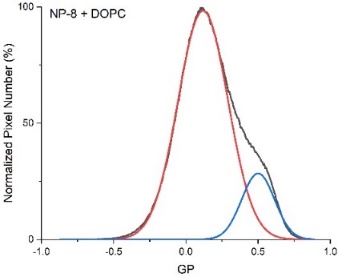** |
| **(c)** | **HepG-2 Cells** | |
| **Blank** | 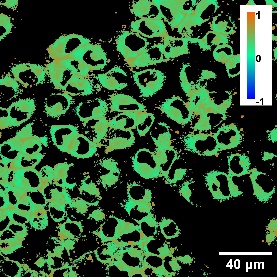 | **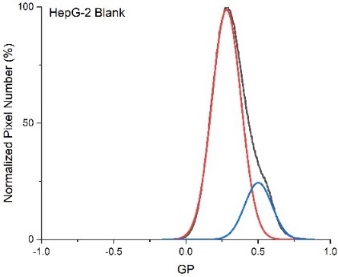** |
| **DOPC Treated** | 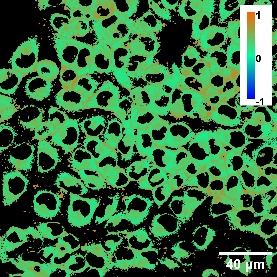 | 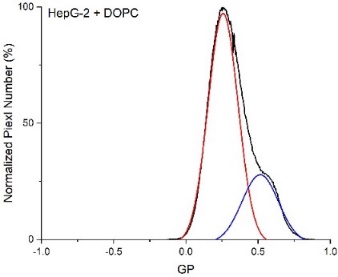 |
| **Figure S-1.** Influence of DOPC liposome treatment on the HepG-2 and NP-8 cell membrane *GP* variations. **(a)** NP-8 and HepG-2 Laurdan *GP* variations after DOPC liposomes treatments for 24/48 h, *GP* values were obtained from fluorescence spectrometer. Error bars represent ± standard deviations, *n*=3. **(b)** *GP* images and *GP* histograms of NP-8 cells with and without DOPC liposomes treatments. **(c)** *GP* images and *GP* histograms of HepG-2 cells with and without DOPC liposomes treatments. Cells were treated with 100 μM DOPC liposomes for 24 h. Images were obtained at 37 ℃. Scale bars represent 40 μm. | | |

1. ***GP* histograms deconvolution results**

**Table S-2. Two Gauss distributions of deconvoluted *GP* histograms**

| **Cell** | **Treatment** | **Low *GP* Peak center** | **FWHM 1** | **High *GP* Peak center** | **FWHM 2** | **High *GP* coverage (%)** |
| --- | --- | --- | --- | --- | --- | --- |
| **NP-8** | Blank | 0.116 ± 0.015 | 0.394 ± 0.048 | 0.481 ± 0.024 | 0.291 ± 0.023 | 20.54 ± 6.98 |
|  | DOPC liposome | 0.128 ± 0.041 | 0.427 ± 0.112 | 0.476 ± 0.043 | 0.336 ± 0.044 | 24.25 ± 9.18 |
|  | 9-1 liposome | 0.310 ± 0.037 | 0.630 ± 0.021 | 0.596 ± 0.028 | 0.406 ± 0.027 | 50.49 ± 6.43 |
|  | 7-3 liposome | 0.266 ± 0.022 | 0.679 ± 0.018 | 0.578 ± 0.021 | 0.414 ± 0.027 | 45.27 ± 2.68 |
|  | 5-5 liposome | 0.223 ± 0.023 | 0.678 ± 0.014 | 0.531 ± 0.026 | 0.447 ± 0.016 | 52.95 ± 3.00 |
|  | Free 2-OHOA | 0.155 ± 0.015 | 0.710 ± 0.019 | 0.475 ± 0.008 | 0.487 ± 0.012 | 58.93 ± 1.88 |
| **HepG-2** | Blank | 0.279 ± 0.030 | 0.237 ± 0.011 | 0.500 ± 0.058 | 0.277 ± 0.060 | 22.32 ± 7.20 |
|  | DOPC liposome | 0.285 ± 0.038 | 0.244 ± 0.016 | 0.524 ± 0.058 | 0.251 ± 0.063 | 21.67 ± 10.11 |
|  | 9-1 liposome | 0.359 ± 0.020 | 0.295 ± 0.027 | 0.593 ± 0.007 | 0.217 ± 0.015 | 27.80 ± 7.01 |
|  | 7-3 liposome | 0.325 ± 0.015 | 0.323 ± 0.056 | 0.609 ± 0.026 | 0.261 ± 0.022 | 22.63 ± 2.13 |
|  | 5-5 liposome | 0.335 ± 0.024 | 0.297 ± 0.021 | 0.589 ± 0.003 | 0.221 ± 0.019 | 26.07 ± 4.08 |
|  | Free 2-OHOA | 0.313 ± 0.013 | 0.296 ± 0.019 | 0.594 ± 0.022 | 0.243 ± 0.019 | 25.03 ± 4.71 |

NP-8 and HepG-2 cells were imaged at 37°C. For the treatment groups, cells were incubated for 24 h with media containing DOPC liposome (100 μM), 2-OHOA-embedded liposome (containing 100 μM 2-OHOA) or free 2-OHOA (100 μM). For blank control group and each treatment group, 3 replicate plates of cell samples were stained with Laurdan and imaged. 3 to 5 images were obtained from each plate of cells, with each image generated by averaging 4 scanning frames. The obtained *GP* histograms of *GP* images, were deconvoluted into two Gauss distributions. The peak center, full width at half maximum (FWHM), and percentage of pixels associated with high *GP* peak (High *GP* coverage) were averaged over 5-8 images.

1. **Comparison of the influence of 2-OHOA-embedded liposomes and free 2-OHOA**

| **(a)** | **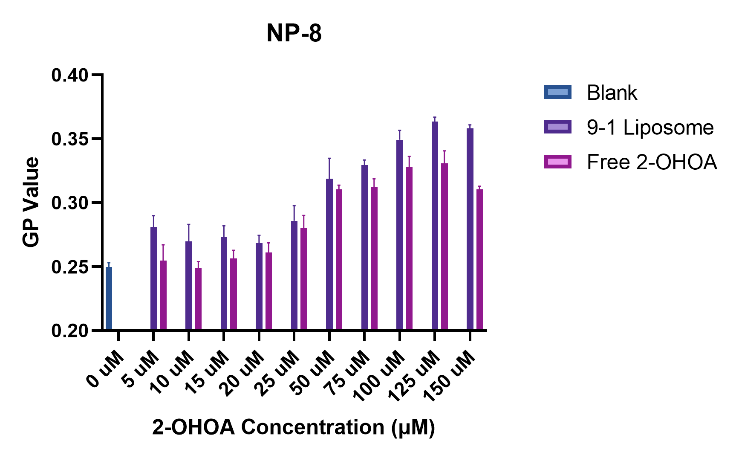** |
| --- | --- |
| **(b)** | **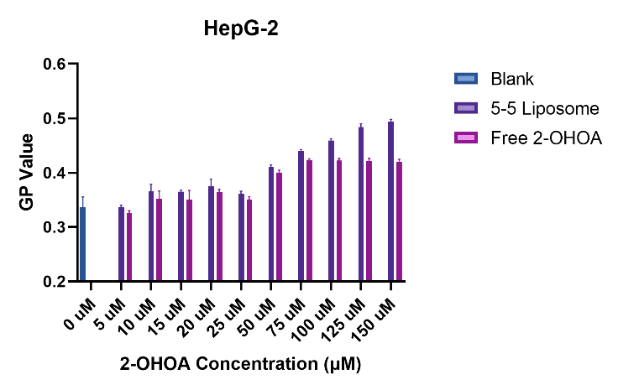** |
| **Figure S-2.** Comparation of the influence of liposome formulation and free 2-OHOA on the cell membrane *GP* value variations. **(a)** NP-8 cell *GP* values after 24 h treatment using 9-1 liposomes and free 2-OHOA at different concentrations; **(b)** HepG-2 cell *GP* values after 24 hours treatment using 5-5 liposomes and free 2-OHOA at different concentrations. the *GP* values were obtained using fluorescence spectrometer (measured at 37°C, excitation wavelength was set at 345 nm, emission spectra were collected from 400 to 600 nm), *GP* values were calculated according to the equation described in experimental section. Error bars represent ± *s.d*. (*n*=3‒5) | |

1. **Lipid droplet staining and observation**

| **(a)** | **NP-8** | | **HepG-2** |
| --- | --- | --- | --- |
| **Blank** | 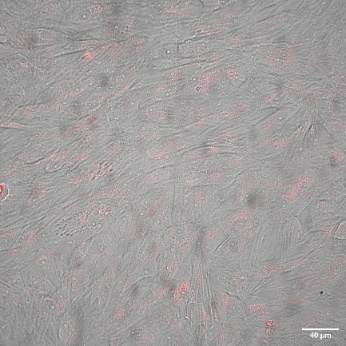 | 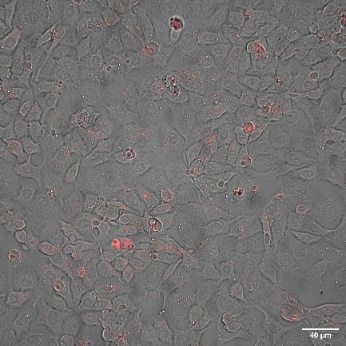 | |
| **2-OHOA Treated** | 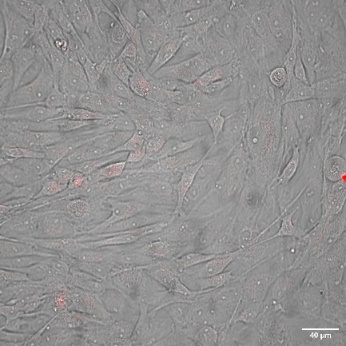 | 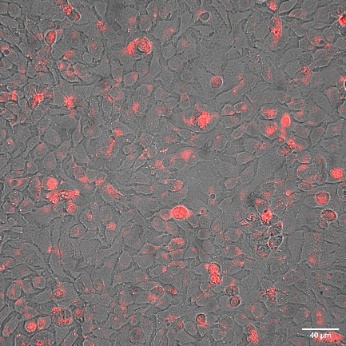 | |
| **(b)** | **Blank Control** | **2-OHOA Treated** | |
| **Bright Field** | 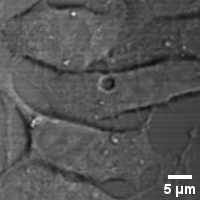 | 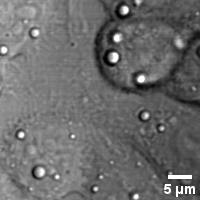 | |
| ***GP* Images** | 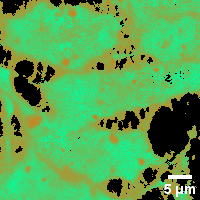 | 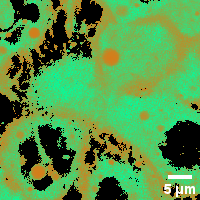 | |
| **Figure S-3.** Lipid droplets images. **(a)** Fluorescence microscopy images of Lipi-Red stained NP-8 and HepG-2 cells. The shown images are merged from bright field image and red channel image. Magnification was 40 times. Scarle bars represent 40 μm. **(b)** Bright field and *GP* images of HepG-2 with and without 2-OHOA treatments, scale bars represent 5 μm. For the treatment group, cells were incubated with 100 μM 2-OHOA for 24 h before staining and imaging. | | | |

1. **Illustration of endocytosis-dependent enhancement of 2-OHOA liposome performance**

**
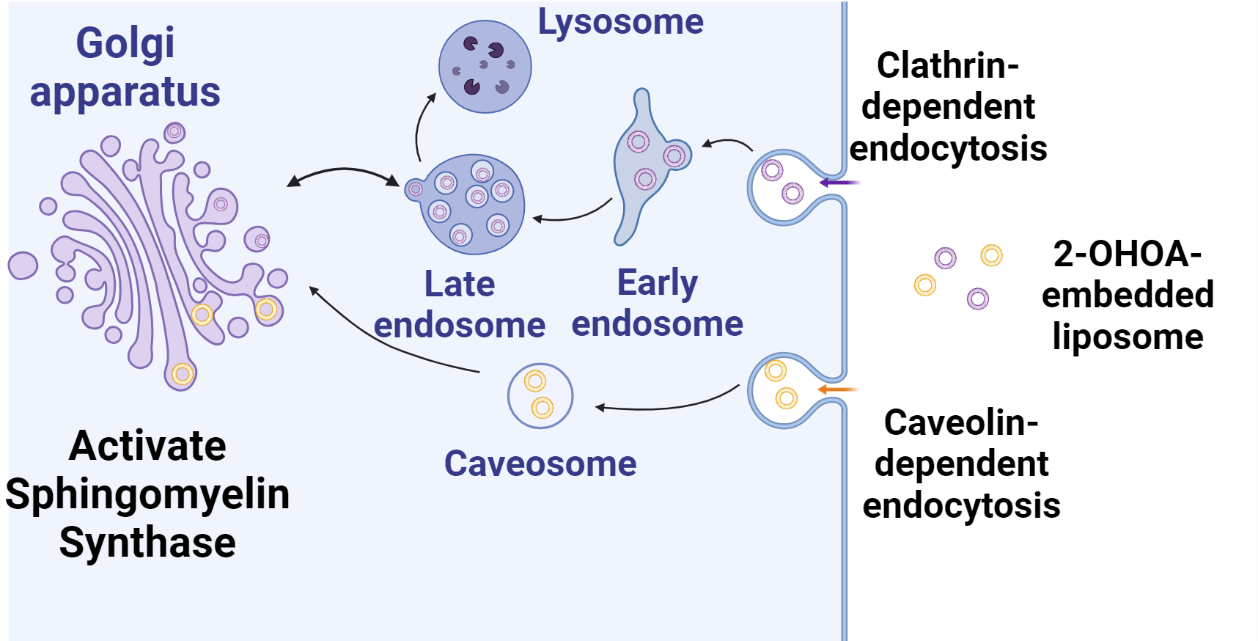
**

| **Figure S-4.** Schematic illustration depicts a hypothetical scenario of the endocytosis process of 2-OHOA-embedded liposomes, enhancing the impact of 2-OHOA on cancer cells. |
| --- |

1. **MTT Assay**

| 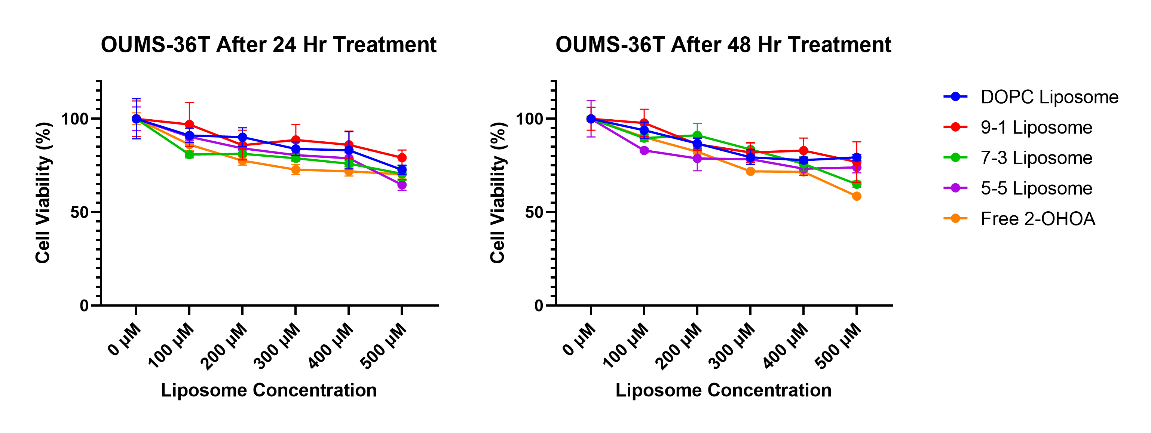 |
| --- |
| **Figure S-5.** MTT assay results. OUMS-36T cells were treated with different liposomes as wells as free 2-OHOA at different concentrations for 24 h and 48 h. Error bars represent ± *s.d*, *n*=3. |

1. **Anticancer performance of 2-OHOA embedded liposome**

| **(a)** | **Blank** | **9-1 Liposome** | **Free 2-OHOA** |
| --- | --- | --- | --- |
| **NP-8** | **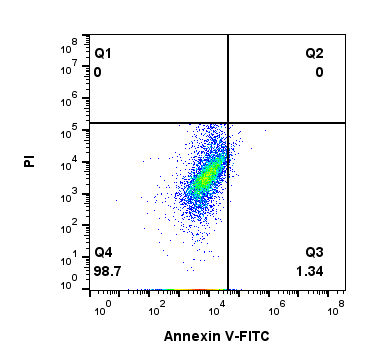** | **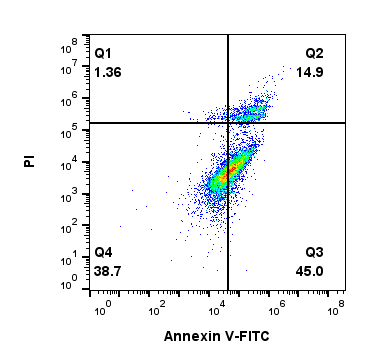** | **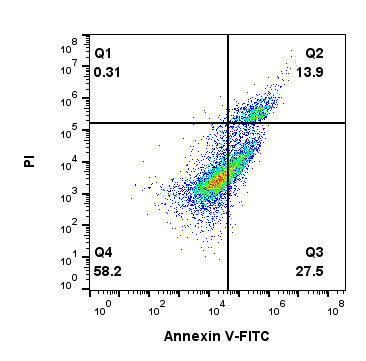** |
| **(b)** | **Blank** | **5-5- Liposome** | **Free 2-OHOA** |
| **HepG-2** | **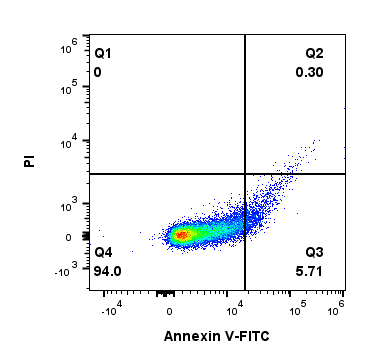** | **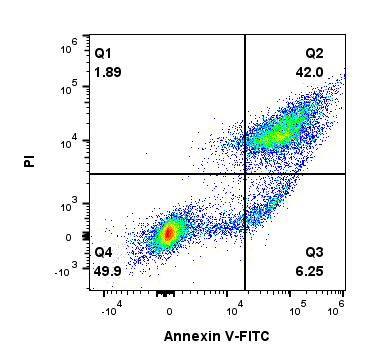** | **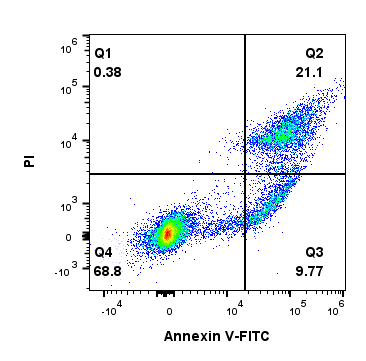** |
| **(c)** | **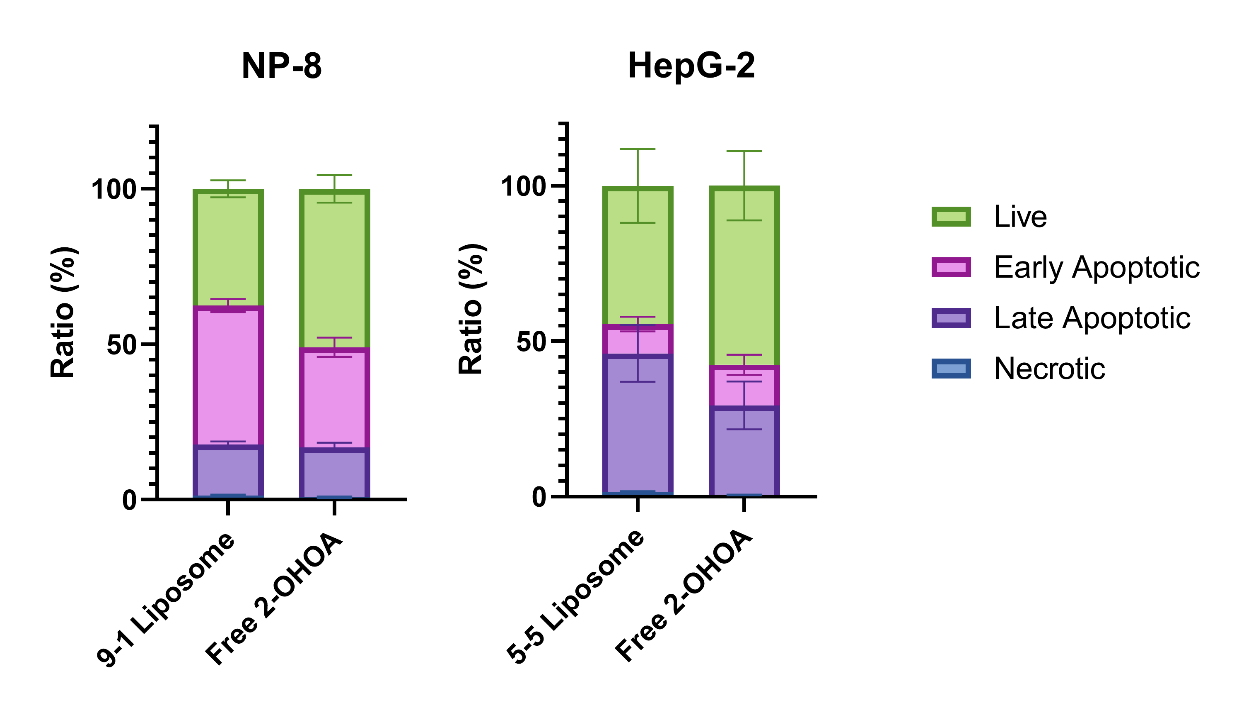** | | |
| **Figure S-6.** Apoptosis assay result. Annexin-V/PI assay flow cytometry result dot graph of **(a)** NP-8 cells and **(b)** HepG-2 cells. **(c)** Summarized apoptotic ratio of NP-8 and HepG-2 cells after treatments. NP-8 cells were treated for 48 h with 9-1 liposome (containing 100 μM 2-OHOA) or free 2-OHOA (100 μM); HepG-2 cells were treated for 48 h with 5-5 liposome (containing 100 μM 2-OHOA) or free 2-OHOA (100 μM). Error bars represent ± *s.d* (*n*=3). | | | |

1. **G-factor calibration of GP images using a reference solution**

| 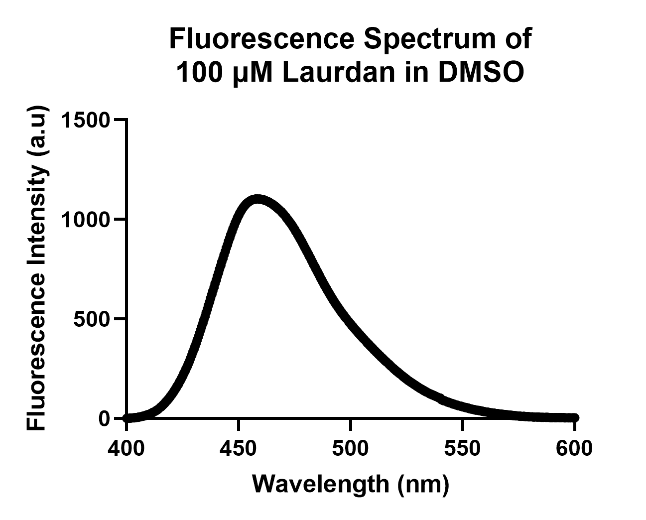 |
| --- |
| **Figure S-7**. Fluorescence spectrum of Laurdan (100 μM) in DMSO. Data was obtained using fluorescence spectrometer (FP-8500, Jasco, Japan). The excitation was set as 345 nm, and emission fluorescence intensity was measured from 400 to 600 nm wavelength. |

Laurdan (100 μM) in DMSO was used as the reference solution for calibration. Laurdan fluorescence spectrum in reference solution was measured using fluorescence spectrometer (FP-8500, Jasco, Japan) with an excitation light of 345 nm wavelength. The reference *GP* value (${GP}_{r}$) was calculated according to equation (S-1).

$\boldsymbol{GP}_{\boldsymbol{r}}\boldsymbol{=}\frac{\boldsymbol{(}\boldsymbol{I}_{\boldsymbol{440}}\boldsymbol{-}\boldsymbol{I}_{\boldsymbol{490}}\boldsymbol{)}}{\boldsymbol{(}\boldsymbol{I}_{\boldsymbol{440}}\boldsymbol{+}\boldsymbol{I}_{\boldsymbol{490}}\boldsymbol{)}}$ **(S-1)**

where $I_{440}$ and $I_{490}$ represent the Laurdan fluorescence intensity at wavelengths of 440 nm and 490 nm, respectively.

The reference solution was imaged using two-photon microscopy, and the fluorescence images of the blue and cyan channels were obtained, and the Laurdan calibration factor ($G_{Laurdan}$) was calculated according to equation (S-2).

$\boldsymbol{G}_{\boldsymbol{Laurdan}}\boldsymbol{=}\frac{\boldsymbol{I}_{\boldsymbol{b}\boldsymbol{lue}}\boldsymbol{\times(}\boldsymbol{1}{\boldsymbol{-}\boldsymbol{GP}}_{\boldsymbol{r}}\boldsymbol{)}}{\boldsymbol{I}_{\boldsymbol{cyan}}\boldsymbol{\times(}\boldsymbol{1}{\boldsymbol{+}\boldsymbol{GP}}_{\boldsymbol{r}}\boldsymbol{)}}$ **(S-2)**

Where $I_{blue}$ is the is the Laurdan fluorescence intensity of the blue channel and $I_{cyan}$ is the Laurdan fluorescence intensity of the cyan channel;

The cell *GP* images were obtained according to equation (S-3).

$\boldsymbol{GP}_{\boldsymbol{m}}\boldsymbol{=}\frac{\boldsymbol{I}_{\boldsymbol{blue}}\boldsymbol{-}\boldsymbol{(}\boldsymbol{G}_{\boldsymbol{Laurdan}}\boldsymbol{\times}\boldsymbol{I}_{\boldsymbol{cyan}}\boldsymbol{)}}{\boldsymbol{I}_{\boldsymbol{blue}}\boldsymbol{+(}\boldsymbol{G}_{\boldsymbol{Laurdan}}\boldsymbol{\times}\boldsymbol{I}_{\boldsymbol{cyan}}\boldsymbol{)}}$  **(S-3)**

Where ${GP}_{m}$ is the *GP* value calculated using the tow-photon microscopy images; $I_{blue}$ is the Laurdan fluorescence intensity of the blue channel and $I_{cyan}$ is the Laurdan fluorescence intensity of the cyan channel; $G_{Laurdan}$ is the Laurdan calibration factor.

1. **High-performance thin film chromatography (HPTLC) results**


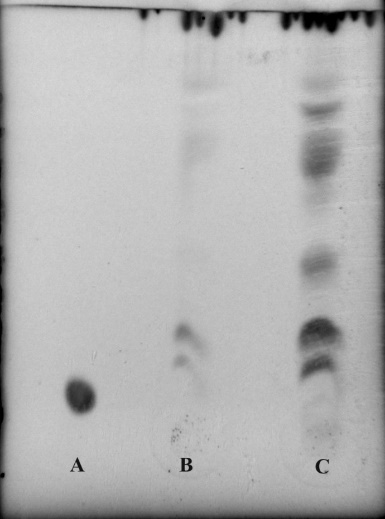


**Figure S-8.** NP-8 cell membrane lipid HPTLC results. **A**: brain sphingomyelin standard sample (1 mg/mL); **B**: extracted lipid from NP-8 cells (no treatment; phospholipid lipid 1 mg/mL); **C**: extracted lipid from NP-8 cells (treated for 24 h using 100 μM 2-OHOA; phospholipid lipid 1 mg/mL).
